# Serum supplementation during bovine embryo culture affects their development and proliferation through macroautophagy and endoplasmic reticulum stress regulation

**DOI:** 10.1371/journal.pone.0260123

**Published:** 2021-12-09

**Authors:** Edgar Joel Soto-Moreno, Ahmed Balboula, Christine Spinka, Rocío Melissa Rivera

**Affiliations:** 1 Division of Animal Sciences, University of Missouri, Columbia, MO, United States of America; 2 College of Agriculture, Food and Natural Resources, University of Missouri, Columbia, MO, United States of America; Colorado State University, UNITED STATES

## Abstract

Serum supplementation during bovine embryo culture has been demonstrated to promote cell proliferation and preimplantation embryo development. However, these desirable outcomes, have been associated with gene expression alterations of pathways involved in macroautophagy, growth, and development at the blastocyst stage, as well as with developmental anomalies such as fetal overgrowth and placental malformations. In order to start dissecting the molecular pathways by which serum supplementation of the culture medium during the preimplantation stage promotes developmental abnormalities, we examined blastocyst morphometry, inner cell mass and trophectoderm cell allocations, macroautophagy, and endoplasmic reticulum stress. On day 5 post-insemination, > 16 cells embryos were selected and cultured in medium containing 10% serum or left as controls. Embryo diameter, inner cell mass and trophectoderm cell number, and macroautophagy were measured on day 8 blastocysts (BL) and expanded blastocysts (XBL). On day 5 and day 8, we assessed transcript level of the ER stress markers *HSPA5*, *ATF4*, *MTHFD2*, and *SHMT2* as well as *XBP1* splicing (a marker of the unfolded protein response). Serum increased diameter and proliferation of embryos when compared to the no-serum group. In addition, serum increased macroautophagy of BL when compared to controls, while the opposite was true for XBL. None of the genes analyzed was differentially expressed at any stage, except that serum decreased *HSPA5* in day 5 > 16 cells stage embryos. *XBP1* splicing was decreased in BL when compared to XBL, but only in the serum group. Our data suggest that serum rescues delayed embryos by alleviating endoplasmic reticulum stress and promotes development of advanced embryos by decreasing macroautophagy.

## Introduction

*In vitro* production (IVP) of bovine embryos is a common practice in the livestock industry, contributing to over one million transferrable embryos in 2019 [1]. On average, blastocyst rates (blastocyst/oocyte) of IVP embryos at day seven or eight post-fertilization usually fluctuates between 30 and 40 percent [2–5]. Various factors and protein sources have been used to supplement IVP media in order to promote embryo development *in vitro* [6–11]. For example, bovine serum (e.g. fetal bovine serum, estrus cow serum) is commonly used as a component of the oocyte maturation medium [6–8] and has been used to supplement the embryo culture medium [12, 13]. Studies have shown that the presence of serum during bovine embryo culture promotes blastocyst development and hatching [14, 15] as well as increases total cell number [16]. Further, supplementation of serum starting on the fifth day of culture also results in ~20% more blastocysts by day seven, when compared to the unsupplemented group [13]. Although these are desirable outcomes, serum has also been associated with increased intracellular lipid droplet accumulation [17], decreased tolerance to cryopreservation [17, 18], and developmental anomalies such as fetal overgrowth and placental malformations [19–22], suggesting that one or more of its components alter the molecular mechanisms involved in conceptus growth and development. In fact, the addition of serum during bovine in vitro culture (IVC) has been shown to alter gene expression of pathways involved in autophagy, growth, and development in blastocyst stage embryos when compared to controls [18, 23, 25]. Although serum effects on bovine blastocyst development, total cell number, and gene expression have been studied, there is no information regarding the effect of this supplement on the autophagic activity of bovine embryos.

Autophagy is an evolutionary conserved mechanism, by which the cell mediates degradation and recycling of cytoplasmic cargo. In macroautophagy, double-membraned autophagosomes are formed *de novo* from phagophores and enclose cargo that is destined to be degraded and/or recycled [24]. The autophagosome then fuses with the lysosome and forms the autophagolysosome complex to degrade the cargo. Macroutophagy has been shown to have many roles in mammalian preimplantation embryo development. During the oocyte-to-embryo transition, maternal proteins, mRNAs, and organelles are degraded through autophagic activity while zygotic mRNAs and proteins are synthesized [25]. This is suggested to provide amino acids, energy, and components for further embryonic development and for newly synthetized proteins to emerge from the embryo [25]. In accordance with this, the inhibition of macroautophagy (by supplementation of 3-methyladenine (3MA) during *in vitro* maturation) reduced the percentage of embryos that cleaved and formed day eight blastocysts, when compared to controls [26]. These results support the notion that mammalian embryos require macroautophagy-mediated catabolism of maternal proteins, mRNAs, and molecules to carry out mRNA and protein anabolism.

Macroutophagy has also been suggested to have a role in blastocyst cavitation, as mouse embryoid bodies null for the key macroautophagy regulator Beclin-1 showed decreased cystic bodies and expansion, when compared to wildtype controls [27]. Under normal conditions, macroautophagy is constitutively active at low levels, only degrading excess or unnecessary biomolecules (e.g. proteins, lipids, dysfunctional organelles) [24]. Under nutrient starvation, macroautophagy is upregulated in order to recycle necessary and/or unavailable components for cell survival and support [28, 29]. Besides nutrient starvation, other cellular responses to adverse biological processes can trigger autophagosome formation and autophagic activity. For example, excessive protein translation and/or chemical inhibitors (e.g. tunicamycin (TM) and thapsigargin (TH)) can lead to accumulation of primary proteins in the ER, where the proteins have to be folded [30, 31]. Failure of proper protein folding accumulates them in the organelle and leads to ER stress [32], which activates the unfolded protein response (UPR) [32, 33] by triggering the heat shock protein family A member 5 (HSPA5) [34]. HSPA5 gene expression increases during ER stress [35], leading to the activation of the UPR [36] by activating the luminal domain-bound sensor proteins: activating transcription factor 6 (ATF6), endoplasmic reticulum to nucleus signaling 1 (ERN1/IRE-1), and eukaryotic translation initiation factor 2 alpha kinase 3 (EIF2AK3/PERK) [32, 33]. When these sensor proteins are activated, downstream signaling cascades mediate control mechanisms that promote ER homeostasis by adaptive (i.e. ER protein folding, macroautophagy, endoplasmic reticulum associated degradation (ERAD)) or pro-apoptotic responses [31, 37]. During ER stress, ERN1/IRE-1 RNase splices the X-box binding protein 1 (XBP1) mRNA in the fourth exon [38, 39], which translates to a transcription factor that promotes ERAD and proteostasis [40]. The levels of the unspliced and spliced forms of *XBP1* mRNA have been showed to serve as a molecular marker to determine ER stress and UPR [38]. During acute ER stress, EIF2AK3/PERK initiates downstream signaling to the UPR-associated chaperone and the activating transcription factor 4 (ATF4), which promotes expression of pro-apoptotic genes [41, 42]. Similar to unspliced and spliced *XBP1*, increased ATF4 mRNA levels have also been associated to chemically induced ER stress [43]. In addition, a recent report identified increased transcription of the increased UPR-associated genes (methylenetetrahydrofolate dehydrogenase (NADP+ dependent) 2 methenyltetrahydrofolate cyclohydrolase (MTHFD2) and serine hydroxymethyltransferase 2 (SHMT2)) when ER stress was chemically induced (TM and TH) in HEK293 cells [44]. Thus, molecular markers for the UPR response can be detected at the mRNA level as well as in protein levels. Taken together, the aforementioned studies point to various roles of macroautophagy in mammalian preimplantation embryo development, from physiological events to nutrient availability responses. Thus, the supplementation of serum to the culture medium of bovine embryos could decrease excessive macroautophagy due to nutrient insufficiency by providing extracellular nutrients but can also alter molecular mechanisms that may lead to long-term developmental anomalies after implantation.

In this study, we tested the hypothesis that bovine embryos cultured in the presence of serum have increased protein synthesis, leading to ER stress and the activation of the unfolded protein response, and ultimately leads to increased autophagic activity. For this, we cultured embryos in the presence or absence of 10% estrus cow serum from day five through day eight of development, and performed analysis of morphometry, cell number, autophagic activity, and ER stress-related gene expression. We found that serum increases embryo diameter and total cell number, alleviates ER stress, and affects autophagic activity in a stage of development-specific manner. As a result of these findings, we propose that the addition of serum to the culture medium on day 5 post insemination results in a higher number of embryos reaching the blastocyst stage on day 7 and 8 of development 1) by rescuing developmentally compromised embryos through alterations in autophagy- and ER stress-associated molecular mechanisms, and/or 2) by the dysregulation of molecular pathways involved in growth, such as the Mammalian Target of Rapamycin complex 1 (MTORC1) pathway, thus causing hasten embryonic growth which ultimately result in an embryo with altered development.

## Materials and methods

### *In vitro* production of embryos and experimental design

All chemicals were purchased from Sigma-Aldrich (St Louis, MO), unless otherwise indicated. All in vitro production (IVP) procedure media were purchased from Caisson Laboratories, unless otherwise indicated. Bovine oocytes derived from crossbred beef cattle were purchased from DeSoto Biosciences, Inc. (Seymour, TN) and Simplot (Fresno, CA). *In vitro* production of bovine embryos was performed as previously described by us [20]. Briefly, cumulus oocyte complexes (COCs) were shipped overnight from DeSoto Biosciences, Inc. or Simplot at 38.5°C in oocyte maturation medium. After 21 hours of maturation, COCs were washed twice in HEPES-TALP (3mg/mL bovine serum albumin- fraction V, 10μl/mL sodium pyruvate, and 1.5μL/mL gentamicin) medium and once in IVF-TALP (6mg/mL bovine serum albumin- essential fatty acid free, 10μl/mL sodium pyruvate, 0.5μL/mL gentamicin, and 5μL/mL heparin) medium. COCs were then transferred in groups of ~50 to 600μL of IVF-TALP medium. For *in vitro* fertilization (IVF), conventionally frozen Angus bull semen (NAAB code: 29AN1922) was thawed at 38.5°C and purified by density centrifugation (Irvine Scientific, Santa Ana, CA) at 1500 x g for five minutes. Purified sperm cells were washed in HEPES-TALP medium once and centrifuged at 195 x g for five minutes. The sperm pellet was collected and diluted with IVF-TALP medium. Co-incubation of COCs and sperm cells was carried out for 18 hours at 38.5°C in a humidified atmosphere containing 5% CO_2_. Putative zygotes were stripped of their cumulus cells by vortexing for 3.5 minutes in 100μL of HEPES-TALP medium, washed, and cultured in 500μL of modified potassium simplex optimized medium (KSOM) at 38.5°C in a humidified atmosphere containing 5% CO_2_, 5% O_2_ and 90% N_2_. At 120 hours post insemination (hpi; day 5), > 16 cells embryos were pooled, divided and transferred to fresh KSOM (S1 Fig). For the serum group, 50μL of estrus cow serum was added to the medium (KSOM-S). The serum was generated in house by collecting blood from 24 crossbred beef heifers (primarily Angus) at estrus. The control group remained unsupplemented (KSOM-NS). Embryos were returned to the incubator and cultured for an additional 68 hours (day 8), at which time they were separated by stage into blastocysts (BL) or expanding/expanded blastocysts (XBL) based on their size and the thickness of their zona pellucida when compared to arrested cleavage stage embryos. Embryos were either processed immediately or stored individually at -86°C (S1 Fig). These procedures were repeated at least three times on different days.

### Live-cell imaging microscopy

To determine autophagosome formation (indirect measure of autophagic activity), 54 KSOM-NS (BL = 32, XBL = 22) and 75 KSOM-S (BL = 30, XBL = 45) day eight embryos were washed in fresh unsupplemented KSOM to which 1mg/mL polyvinylpyrrolidone (KSOM-PVP) had been added to prevent embryos from sticking to the bottom of the dish. Embryos were then transferred to wells containing 250μL of KSOM-PVP containing 0.002% of CYTO-ID^®^ autophagosome formation fluorescence label solution (Autophagy detection kit; ENZO Life Sciences Inc, ENZ51031 Farmingdale, NY) and incubated for 25 minutes at 38.5°C in a humidified atmosphere containing 5% CO_2_. After incubation, the embryos were transferred to 5μL drops of KSOM-PVP on a glass-bottom imaging dish and covered with mineral oil (MatTek, Ashland, MA). Bright field and fluorescence imaging were taken using a DMi8 S Platform inverted microscope and LAS X software (Leica Microsystems Inc, Buffalo Grove, IL) at 40X immersion oil magnification. Mean gray value for autophagosome formation was normalized to the fluorescence exposure intensity during live imaging and embryo area (excluding the zona pellucida; ZP). ZP thickness and average embryo diameter were measured (in micrometers; μm). Data were analyzed using FIJI software (ImageJ, NIH).

### Inner cell mass and trophectoderm cell count

Day eight KSOM-NS (18 BL and 8 XBL) and KSOM-S (21 BL and 18 XBL) embryos were fixed for 20 minutes in 4% paraformaldehyde followed by three washings in wash buffer (1X PBS + 0.1% Tween 20 and 0.1% bovine serum albumin (BSA)). Fixed embryos were permeabilized in 100μL of 1X PBS + 0.5% Triton X-100 for 30 minutes then blocked in 100μL of 1X PBS + 5% BSA at room temperature for one hour, followed by three washings in 100μL of wash buffer. Embryos were then incubated overnight in 50μL of ready-to-use CDX2 mouse monoclonal antibody (caudal type homeobox 2; BioGenex, Fremont, CA). CDX2 is a protein primarily localized in the trophectoderm (TE) in bovine blastocyst [45]. After incubation in primary antibody, embryos were washed three times before incubating them for one hour at room temperature in goat anti-mouse IgG Alexa Fluor® 555 secondary antibody (Abcam, Cambridge, MA). DNA was subsequently labelled by incubating embryos for 10 minutes at room temperature in 50μL of Hoescht 33342 (1μg/mL) in 1X PBS followed by three washings in sterile water. Finally, processed embryos were mounted in 15μL of Fluoromount™ Aqueous Mounting Medium on microscope slides and their TE and total cell number visualized using a Leica DM5500 B Fluorescence Motorized Phase Contrast Microscope (Leica Microsystems Inc, Buffalo Grove, IL) and counted using FIJI software.

### RNA isolation and complementary DNA (cDNA) synthesis of bovine embryos

Total RNA from bovine pre-implantation embryos was isolated using the RNAqueous™ Micro Total RNA Isolation kit (Invitrogen, Carlsbad, CA) as per manufacturer’s specifications. Prior RNA isolation, 1000ng of *Zea mays* aquaporin *(NIP3-1)* RNA was added to each sample to use as an exogenous normalizer for nucleic acid losses. Complementary DNA (cDNA) was synthesized from total RNA using the SuperScript™ IV Reverse Transcriptase (Invitrogen, Carlsbad, CA), according to the manufacturer’s instructions. Each sample was diluted to a final volume of 25μL and used for reverse transcriptase polymerase chain reaction (RT-PCR). Pools of two embryos were used for quantitative RT-PCR while single embryos were used for semi-quantitative RT-PCR (i.e. for *XBP1* analysis).

### Quantitative and semi-quantitative RT-PCR

To assess ER stress, quantitative RT-PCR of genes upregulated during the unfolded protein response (UPR; response to ER stress) was done using TaqMan® probes (ThermoFisher Scientific, Hanover Park, IL) using a QuantStudio 3 Real-Time PCR System (Applied BioSystems, Waltham, Massachusetts). *HSPA5* and *ATF4* transcript level were quantified to determine ER stress and UPR response using the Bt03244882_m1 and Bt03221057_m1 TaqMan® probes, respectively. The UPR-dependent expression of the enzymes *MTHFD2* and *SHMT2* were measured using the Bt03247319_m1 and Bt03225783_m1 probes, respectively. The mRNA level of each target transcript was normalized to the geometric mean of three endogenous normalizers, namely splicing factor 3a subunit 1 (SF3A1; Bt03254301_m1), hydroxymethylbilane synthase (*HMBS*; Bt03234763_m1), and histone variant H2A (*H2AZ1*, Bt03216348_g1). Amplifications were performed in at least duplicates (average cycle threshold difference of duplicates/triplicates for all amplifications = 0.331 ± 0.231). All measured transcripts were initially normalized for procedural losses to the exogenous *Zea mays Nip3a* (Zm04057741_m1). Each group’s cycle threshold difference and 2^Delta-Delta Ct^ was calculated to determine the fold difference in transcript levels [46].

To determine UPR activity, *XBP1* mRNA and its ER stress-dependent spliced form was done by semi-quantitative RT-PCR (S2 Fig). Previously published intron-spanning oligo primers for spliced and unspliced *XBP1* isoforms were used in order to minimize bias created by potential DNA contamination [39] (S1 Table). In the aforementioned report, the forward primer for each *XBP1* variant was designed to bind specifically to either the unspliced or spliced exon 4 sequence, while the same reverse primer was used for both products. RT-PCR products were resolved in a 10% polyacrylamide gel (S2 Fig). Total *XBP1* levels (unspliced + spliced) were used to normalize the expression and percentage of spliced *XBP1* mRNA and band density was quantified using FIJI software.

### Induction of ER stress on day 5 and day 8 embryos

We hypothesized that embryos would perceive the sudden increase in medium nutrients on day 5 (i.e. serum supplementation) as a stressful event, thereby resulting in increased ER stress. To study this question, we treated embryos with Tunicamycin (TM) on day 5 (the day that serum supplementation of the culture medium takes place) in order to have a positive control for ER stress. For this, > 16 cells embryos (120 hpi) from KSOM-S and KSOM-NS were divided in three groups each and cultured for six hours with TM (5μg–as previously published [47]), in vehicle (0.05% ethanol [EtOH]), or left untreated. Six hours was chosen as this timing was previously demonstrated to induce acute ER stress in mammalian cells [48]. TM is an antibiotic that induces ER stress by interfering N-linked glycosylation of proteins resulting in accumulation of misfolded proteins [49]. Therefore, TM serves as a positive control for acute ER stress. After treatment, embryos were collected, washed three times in 1X Dulbecco’s phosphate-buffered saline (DPBS), and stored in -86°C. These embryos were used to determine transcript levels of *HSPA5*, *ATF4*, MTHFD*2*, and *SHMT2* as well as for *XBP1* splicing analyses.

Transcript levels of *HSPA5*, *ATF4*, *MTHFD2*, and *SHMT2* as well as *XBP1* splicing was assessed in BL and XBL in order to determine if culture in the presence of serum from day five through day eight results in chronic ER stress at the blastocyst stage.

### Statistical analyses

Statistical analyses were done using the SAS software version 9.4 (SAS Institute, Cary, NC). Embryo morphometry, macroautophagy, and *XBP1* results were analyzed through analysis of variance (ANOVA) generalized linear model procedure (PROC GLM) and Fisher Least Significant Difference (LSD) method for multiple comparisons. RT-PCR results were calculated by the mean ± S.E.M. model followed by ANOVA PROC GLM and Fisher’s LSD multiple comparison using each group as an individual data point. P-values lower than 0.05 were considered statistically significant whereas p-values <0.10 were considered as trending toward statistical significance.

## Results

### Effect of serum on embryo morphometry, total cell number, and TE allocation

**Fig 1:**
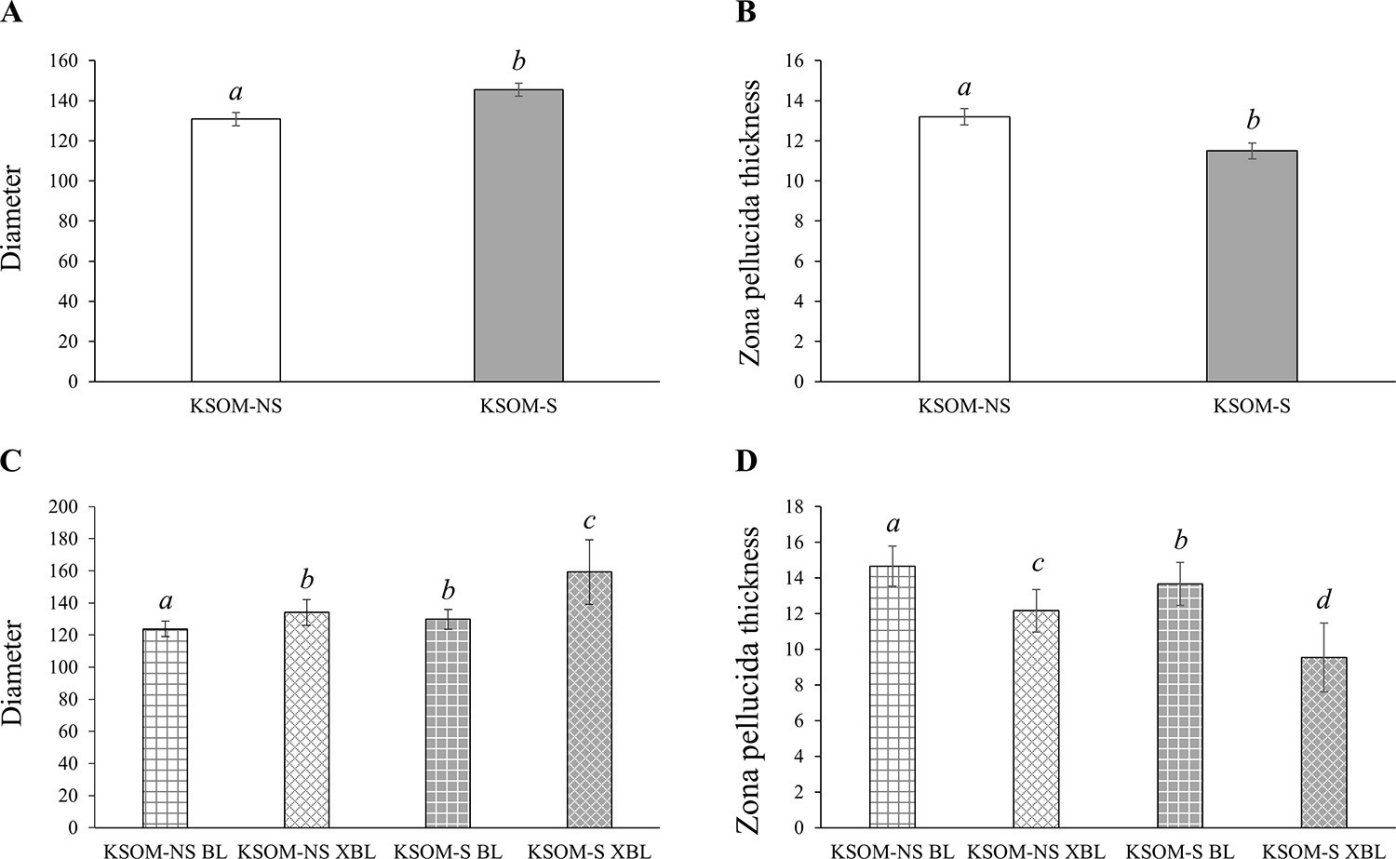
Morphometry of day 8 *in vitro* produced bovine embryos cultured in the presence or absence of estrus cow serum. **A.** Diameter average of total KSOM-NS (white bars) *vs* KSOM-S (grey bars) embryos (*a*, *b* = p<0.0001). **B.** Zona pellucida thickness average of total KSOM-NS (white bars) *vs* KSOM-S (grey bars) embryos (*a*, *b* = p<0.0001). **C.** Diameter average of embryos separated by treatment and stage (*a*, *b*, *c* = p<0.004). **D.** Zona pellucida thickness average of embryos separated by treatment and stage (*a*, *b*, *c*, *d* = p<0.002). Diameter and zona pellucida thickness averages were measured in μm. Number of embryos represented in the bars–KSOM-NS = 54, KSOM-S = 75, KSOM-NS BL = 32, KSOM-NS XBL = 22, KSOM-S BL = 30, and KSOM-S XBL = 45. BL = blastocyst. XBL = expanding/expanded blastocyst. S = Serum. NS = No serum. Shown are averages ± S.E.M.

On average, KSOM-S embryos were larger (p<0.0001; Fig 1A) and had a thinner zona pellucida (p<0.0001; Fig 1B) than KSOM-NS counterparts on day eight of development. When embryo size was compared on a per developmental stage basis, both KSOM-S BL and XBL were bigger than KSOM -NS counterparts (p<0.004; Fig 1C). KSOM-NS XBL were similar in size to the BL from the KSOM-S group, indicating that serum treatment enhances embryonic size (Fig 1C). Both, the BL and XBL of the KSOM-S group had thinner zona pellucida, when compared to similar stage KSOM-NS embryos, also suggesting that serum promotes embryo development (p<0.002; Fig 1D).

**Fig 2:**
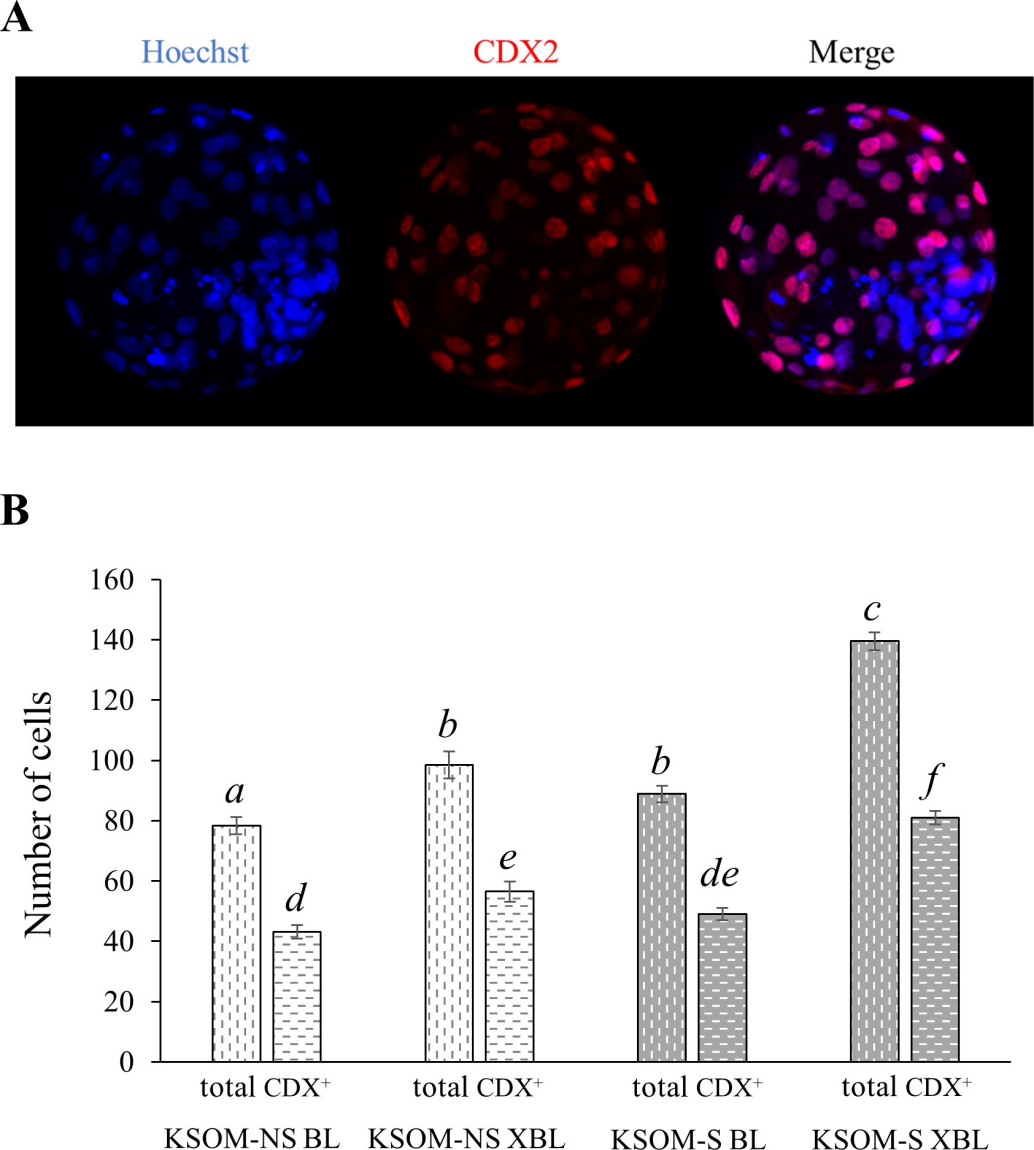
Cell number quantification of *in vitro* produced day 8 bovine embryos cultured in the presence or absence of estrus cow serum. **A.** Immunofluorescence images of a day eight *in vitro* produced bovine embryo. **B.** Total (Hoechst; *a*, *b*, *c* = p<0.011) and trophectoderm (CDX2; *d*, *e*, *f* = p<0.002) cell number averages of KSOM-NS (white bars) *vs* KSOM-S (grey bars) embryos. Number of embryos represented in the bars–KSOM-NS BL = 18, KSOM-NS XBL = 8, KSOM-S BL = 21, and KSOM-S XBL = 18. BL = blastocyst. XBL = expanding/expanded blastocyst. S = Serum. NS = No serum. Shown are averages ± S.E.M.

CDX2 positive cells (presumably TE) and non-CDX2 positive cells (presumably inner cell mass) were identified to determine if serum altered total cell number and cell allocation. As expected, XBL have more (p<0.011) cells than BL (Fig 2). In addition, serum supplementation of bovine blastocysts from day 5 to 8 of culture increased the total number of cells (p<0.002) wherein KSOM-S BL have similar cell number as KSOM-NS XBL (Fig 2). Cell allocation was not altered as a result of serum supplementation.

### Autophagic activity of bovine embryos supplemented with serum during embryo culture

**Fig 3:**
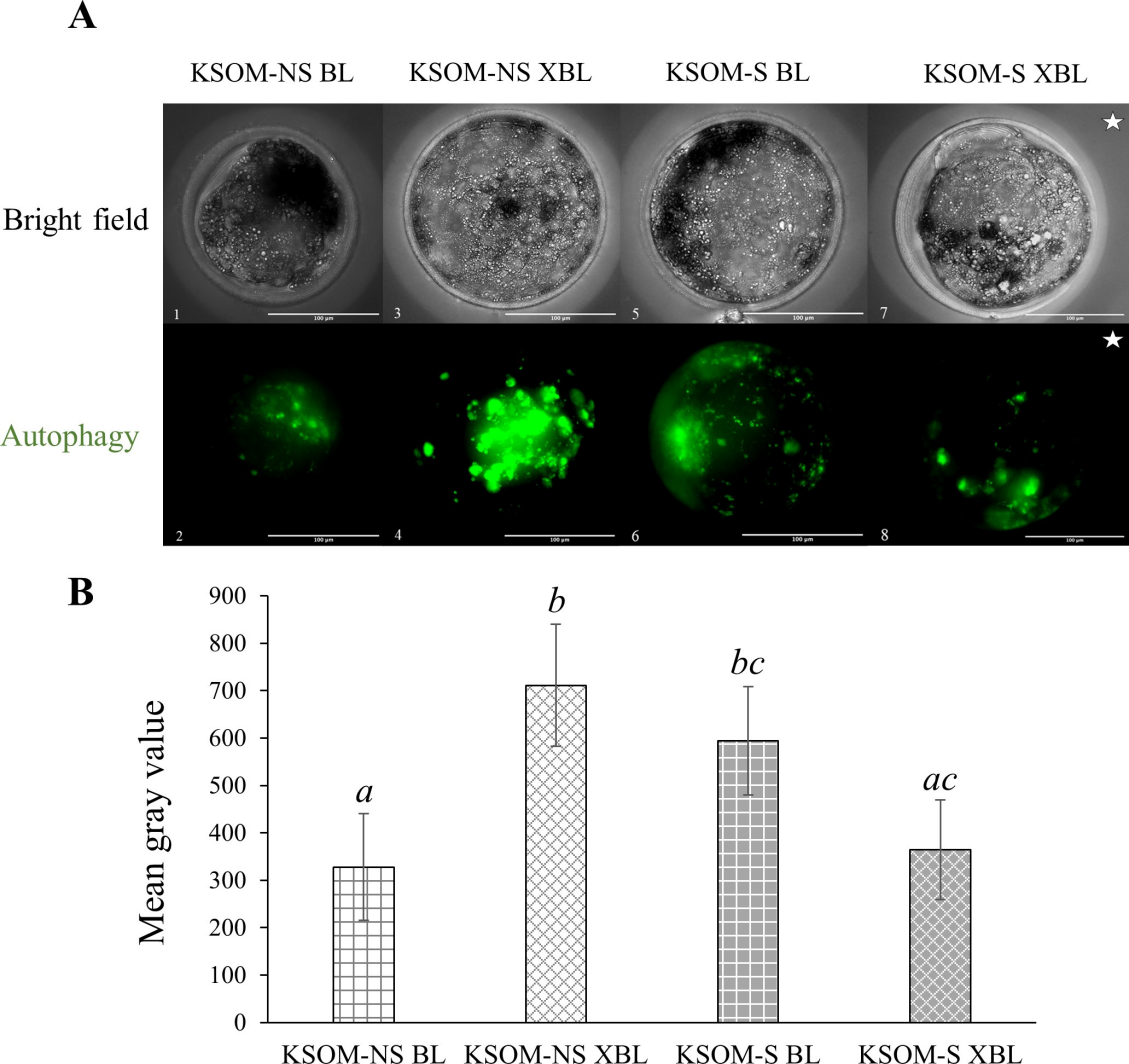
Adjusted macroautophagy of *in vitro* produced bovine embryos cultured in the presence or absence of estrus cow serum. **A.** Live imaging of day eight post-insemination embryos. **B.** Adjusted macroautophagy (mean gray value) of day eight *in vitro* produced KSOM-NS (white bars) *vs* KSOM-S (grey bars) embryos. Bar = 100 μm. *a*, *b*, *c* = p<0.001. **Note**: The embryo identified with a white star (pictures 7 and 8) seems smaller because the original picture was reduced to fit the figure. Number of embryos represented in the bars–KSOM-NS BL = 32, KSOM-NS XBL = 22, KSOM-S BL = 30, and KSOM-S XBL = 45. BL = blastocyst. XBL = expanding/expanded blastocyst. S = Serum. NS = No serum. Shown are averages ± S.E.M.

Autophagic activity was higher (p<0.001) in the KSOM-S BL when compared to KSOM-NS BL, while the opposite (p<0.001) was true when embryos were compared at the XBL stage (Fig 3). In other words, the presence of serum from days five to eight induces a differential effect on autophagic activity in which KSOM-NS BL and KSOM-S XBL have similar lower levels of autophagic activity than KSOM-NS XBL and KSOM-S BL (Fig 3).

### Assessment of ER stress on day 5 and day 8 *in vitro* produced bovine embryos

Day five embryos > 16 cells stage were used to identify if serum induces acute ER stress and/or UPR responses as this is the stage (and day of culture) at which embryos are normally supplemented during *in vitro* culture. Day eight BL and XBL were used to identify the effects of prolonged serum presence during IVC on ER stress and/or UPR responses.

**Fig 4:**
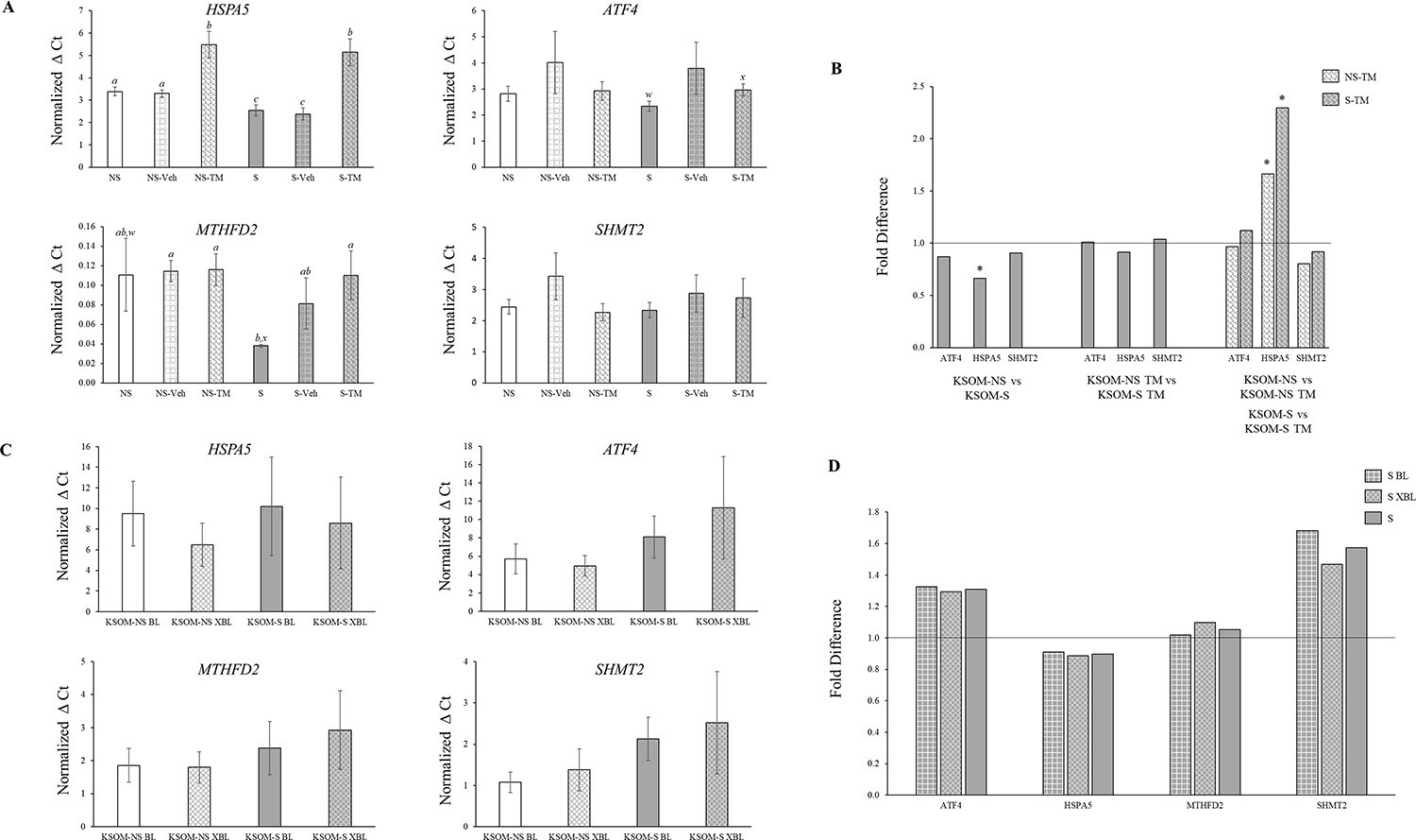
Endoplasmic reticulum stress-associated gene expression between embryos cultured in the presence or absence of estrus cow serum. **A.** Normalized cycle threshold (Ct) of ER stress-associated genes of day five > 16 cells embryos in KSOM-NS (white bars) *vs* KSOM-S (grey bars). Dotted grid pattern = embryos treated with 0.05% EtOH (i.e. vehicle control). Shingle pattern = embryos treated with TM (positive control for ER stress). *a*, *b*, *c* = p<0.001. *w*, *x* = p<0.10. Shown are averages ± S.E.M. **B.** Fold difference of ER stress-related markers of day five > 16 cells embryos cultured in KSOM-NS (white bars) *vs* KSOM-S (grey bars) and treated with TM (shingle pattern). Bars identified with asterisks (*) represent significant fold differences between the group represented by the bar and the group adjusted to a value of 1. * = p<0.03. **C.** Normalized cycle threshold (Ct) of ER stress-associated gene expression of day eight BL (solid bars) and XBL (outlined diamond pattern) in KSOM-NS (white bars) *vs* KSOM-S (grey bars). **D.** Fold difference of ER stress-related markers of BL (large grid pattern) and XBL (diamond pattern) cultured in KSOM-NS (white bars) *vs* KSOM-S (grey bars). Number of embryos represented in the bars–KSOM-NS BL = 9, KSOM-NS XBL = 9, KSOM-S BL = 9, and KSOM-S XBL = 9. BL = blastocysts. XBL = expanding/expanded blastocysts. NS = no serum. S = presence of serum. TM = 5μg Tunicamycin in 0.05% EtOH.

Day 5 embryos were treated with TM in order to induce ER stress (Fig 4A) and to serve as a positive control for the potential effects of serum on acute ER stress. As expected, TM treatment increased *HSPA5* gene expression when compared to untreated groups in both KSOM-NS and KSOM-S embryos (Fig 4A and 4B). No difference was detected in *HSPA5* levels between TM treated embryos from the KSOM-S and KSOM-NS groups (Fig 4A and 4B), indicating saturation of the ER response at the dose used. Contrary to our expectations, > 16 cells stage embryos cultured in KSOM-S showed decreased *HSPA5* when compared to KSOM-NS (Fig 4A and 4B). While expression of *MTHFD2* was detected in 83% of KSOM-NS day five > 16 cells embryos, only 50% of the KSOM-S had detectable levels of this transcript (Fig 4A), indicating low *MTHFD2* expression in the presence of serum (Fig 4A and 4B). Detectable *MTHFD2* in the KSOM-S day five > 16 cells embryos were greater than TM treated embryos and tended to be lower than in KSOM-NS embryos (Fig 4A). No difference was identified for *ATF4* and *SHMT2* gene expression between groups.

At the BL and XBL stages, no differences were observed for any of the genes analyzed between the serum supplemented and unsupplemented groups (Fig 4C and 4D).

**Fig 5:**
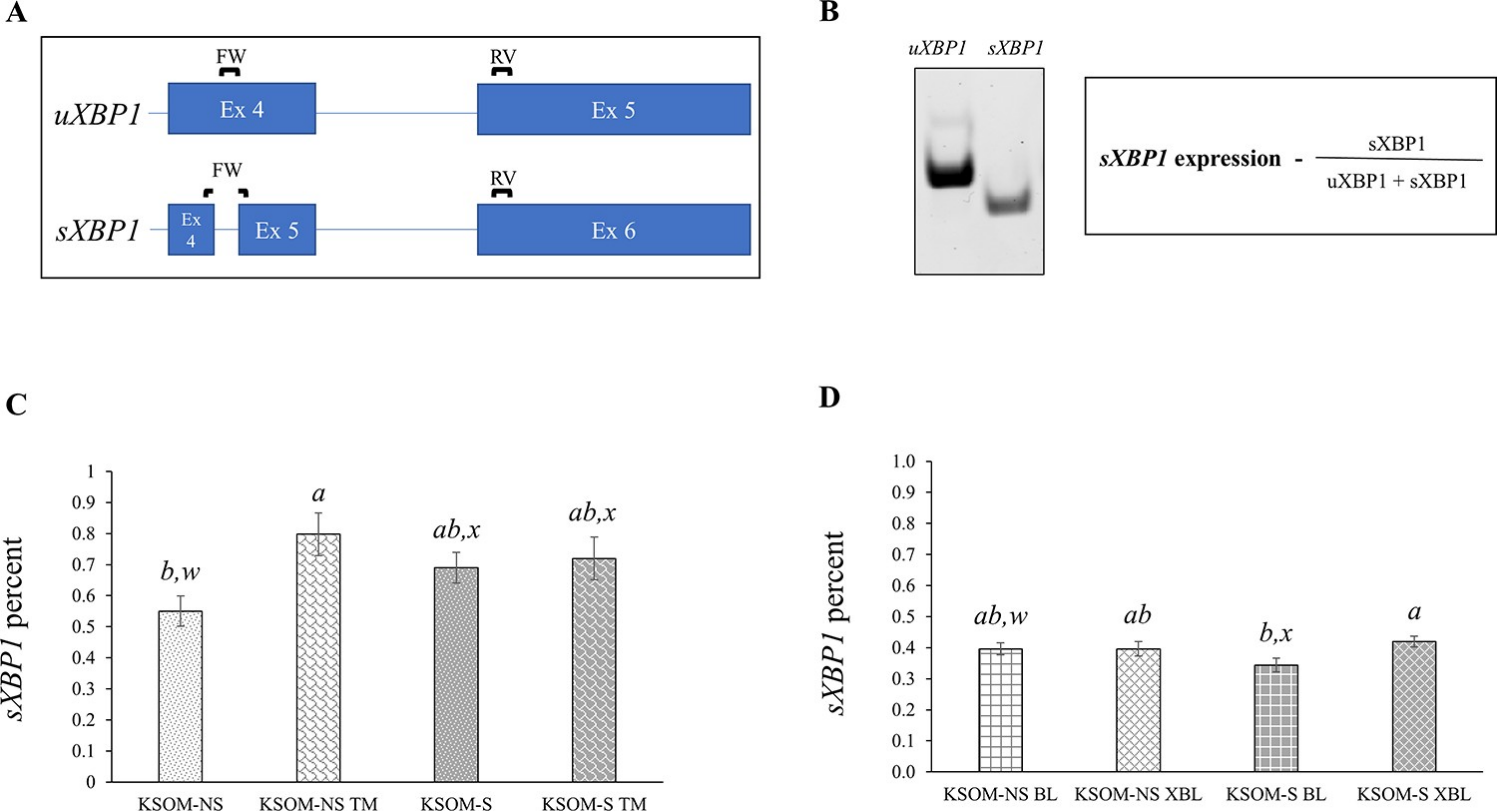
*XBP1* splicing in bovine embryos cultured in the presence of absence of estrus cow serum. **A.** Depiction of intron-spanning primers used to identify *XBP1* variants. Note: the same reverse primer was used to amplify the spliced and unspliced forms of the transcript. **B.** PCR product of *XBP1* variants (left) and formula used (right) to determine the percent of s*XBP1*. **C.** Percent of s*XBP1* in > 16 cells stage embryos cultured in KSOM-NS (white bars) *vs* KSOM-S (grey bars) and treated with TM (shingle pattern). **D.** Percent of s*XBP1* in BL (large grid pattern) and XBL (diamond pattern) cultured in KSOM-NS (white bars) *vs* KSOM-S (grey bars). u*XBP1* = unspliced *XBP1*. s*XBP1* = spliced *XBP1*. Number of embryos represented in the bars–KSOM-NS = 6, KSOM-NS TM = 3, KSOM-S = 6, and KSOM-S TM = 3, KSOM-NS BL = 9, KSOM-NS XBL = 9, KSOM-S BL = 9, and KSOM-S XBL = 9. BL = blastocysts. XBL = expanding/expanded blastocysts. TM = 5μg Tunicamycin. Shown are averages ± S.E.M. *a*, *b* = p<0.02. *w*, *x* = p<0.10.

*XBP1* exon 4 splicing (s*XBP1-* a measure of the unfolded protein response; Fig 5A and 5B) of > 16 cells day five embryos tended to be lower in KSOM-NS, when compared to embryos cultured in the presence of serum (p<0.07, Fig 5C). TM treatment increased the percent of s*XBP1* in the KSOM-NS group when compared to the KSOM-NS untreated embryos (p<0.02, Fig 5C). However, this was not the case for KSOM-S TM-treated embryos (Fig 5C) perhaps indicating mechanism saturation in the presence of serum.

No difference was detected in the percent of total *XBP1* that was spliced between BL and XBL stage embryos in the KSOM-NS group (Fig 5D). BL stage embryos had less s*XBP1* than XBL in the serum group (p<0.02, Fig 5D). BL cultured in the presence of serum tended to have lower levels of s*XBP1* when compared to BL in the KSOM-NS group (p<0.09, Fig 5D), however, no difference in s*XBP1* was observed at the XBL stage between treatment groups (Fig 5D).

## Discussion

In this study, we found that the presence of serum from day five through eight of bovine embryo culture increases embryo diameter and total cell number and causes a stage specific effect on autophagic activity and ER stress. The MTORC1 regulates growth, proliferation, development, and macroautophagy in mammalian cell culture systems [50–54]. MTORC1 signal transduction mechanisms are regulated by insulin [55], growth factors [56], amino acids [57], and glucose [58]. Therefore, we suggest that molecules in serum (i.e. hormones, growth factors, amino acids, and glucose [59, 60]) directly or indirectly alter MTORC1 signaling of embryos cultured in the presence of serum. Future work will address this hypothesis.

An interesting finding of this study, is that the presence of serum from day five through day eight of culture induces a differential effect in embryonic autophagic activity at the blastocyst stage of development; while serum increased macroautophagy of BL, the opposite was true for XBL. We interpret this to mean that the slower developing embryos in the serum group were rescued from arrest by components in serum, and that macroautophagy acts as a survival mechanism perhaps by decreasing apoptosis as previously shown in immortalized IL-3 cells [61]. On the other hand, nutrients in serum may activate MTORC1 signal transduction in the more advanced embryos (i.e. XBL), thus decreasing macroautophagy while at the same time promoting their development. Activation of the MTORC1 leads to decrease macroautophagy by phosphorylation of UNC-51 Like Autophagy Activating Kinase 1 (ULK1; [52]). In addition, removal of MTOR by genetic manipulations led to decreased proliferation of mouse embryos [53]. Another possibility is that the increased total cell number of day eight embryos cultured in the presence of serum is a result of decreased apoptosis in response to components in serum, instead of increased cell proliferation.

We also observed that XBL in the NS group had increased macroautophagy when compared to NS BL. We propose that the increased macroautophagy may be attributed to increased intracellular nutrient recycling [25] to support proper development, proliferation, and growth [24] suggesting that the unsupplemented KSOM medium may be deficient in molecules required for embryos to advance past the BL stage. Induction of macroautophagy has been reported to alleviate ER stress in bovine embryos and increase their development *in vitro* [62]. We suggest that increased levels of macroautophagy in unsupplemented XBL is regulated by nutrient-mediated ER stress and intracellular recycling responses, thereby promoting development and hatching even in the absence of proper amount of nutrients in the medium.

Serum supplementation to the embryo culture medium on day five post-insemination is a practice that has been used to promote blastocyst formation [13, 63]. We wondered how day five > 16 cells-stage embryos would respond to the acute effects of serum supplementation. We analyzed gene expression of ER stress markers six hours after the addition of serum and observed that serum alleviates ER stress as determined by decreased transcript levels of *HSPA5*, a gene linked to pro-survival cellular responses and ER stress response [34]. This observation was contrary to our original hypothesis: that serum would induce acute ER stress. It has been reported that bovine-donor fibroblast cells cultured *in vitro* experience increased ER stress when cultured in serum deprivation [64]. Therefore, it can be speculated that the higher levels of *HSPA5* in the NS day five embryos is the result of a mismatch between the requirement of the embryo and the composition of the culture medium. The observation that TM treatment increased ER stress of day five embryos (as determined by increased *HSPA5* transcript amount), and that their *HSPA5* expression was similar between KSOM-NS and KSOM-S groups suggest that TM saturated the ER stress response in the embryos. By day eight, however, *HSPA5*’s transcript amounts were no different between treatments. This can be interpreted in two ways; first of all, the expression differences between treatment groups were resolved by day eight (i.e., the embryos achieved homeostasis), or secondly, that both embryo culture systems cause equal ER stress.

*XBP1* splicing is a marker of the unfolded protein response (UPR; [38]). The spliced isoform of XBP1 has been shown to directly regulate the expression of pro-survival genes an example being the proto-oncogene MYC [65]. We show here that the UPR of day five embryos (as determined by s*XBP1*) tended to be more active in those embryos which had been supplemented with serum when compared to the NS group. While the UPR activity increased in NS embryos treated with TM, this was not the case for the serum treated embryos suggesting that the UPR was at saturation in this group. On day eight, BL have decreased s*XBP1* and higher macroautophagy than XBL in the serum-supplemented group. This observation led us to propose that serum rescues less developed/ill embryos by alleviating ER stress through the UPR and macroautophagy. In fact, chemically induced macroautophagy with rapamycin (MTORC1 inhibitor) in *in vitro* produced bovine embryos has been shown to decrease ER stress and, in turn, increase development rates [62]. In addition, we further propose that advanced embryos (XBL) in the serum supplemented group have MTORC1 hyperactivity due to components in serum and availability of nutrients, and this in turn increases growth and proliferation through protein anabolism [54]. As mentioned above, induction of macroautophagy has been reported to alleviate ER stress in bovine embryos and increase their development *in vitro* [62] and this coincides with the reduced *sXBP1* in BL cultured in serum in this study, as these embryos also had increased macroautophagy.

This study characterized the morphometry, cell proliferation, autophagic activity, and ER stress-associated gene expression of bovine embryos in relation to serum exposure during culture. Findings suggest an macroautophagy-mediated molecular mechanism by which components in serum alleviate ER stress-mediated s*XBP1* in less fit embryos. This mechanism could explain why serum supplementation leads to increased development rates of embryos cultured *in vitro*. Further research will be needed to determine the involvement of the macroautophagy-ER stress relation in embryo development, to ascertain what components in serum affect the embryos, to characterize which signal transduction mechanisms are altered by the presence of serum, and how components in culture systems could influence long-term embryonic/fetal development and anomalies.

## Supporting information

S1 Table Primer sequence and semi-qPCR program for XBP1 assays. (TIF)

S1 Fig Experimental design. (TIF)

S2 Fig Unspliced, spliced, and total XBP1 semi-quantitative RT-PCR to identify endoplasmic reticulum stress. **A.** Unspliced, spliced, and total XBP1 ratios of single bovine embryos. **B.** Ratio of spliced XBP1 of tunicamycin treated, vehicle, and control embryos. TM treatment indeed shows splicing of XBP1, suggesting an UPR response to induced ER stress. TM = 5μg Tunicamycin. Vehicle = 0.05% ethanol alcohol.
(TIF)

S1 Raw images (PDF)
