# Supplementary figures and images for: Serum supplementation during bovine embryo culture affects their development and proliferation through macroautophagy and endoplasmic reticulum stress regulation
Source: PLoS One. 2021 Dec 9;16(12):e0260123. doi: 10.1371/journal.pone.0260123 (PMC8659681; doi:10.1371/journal.pone.0260123)

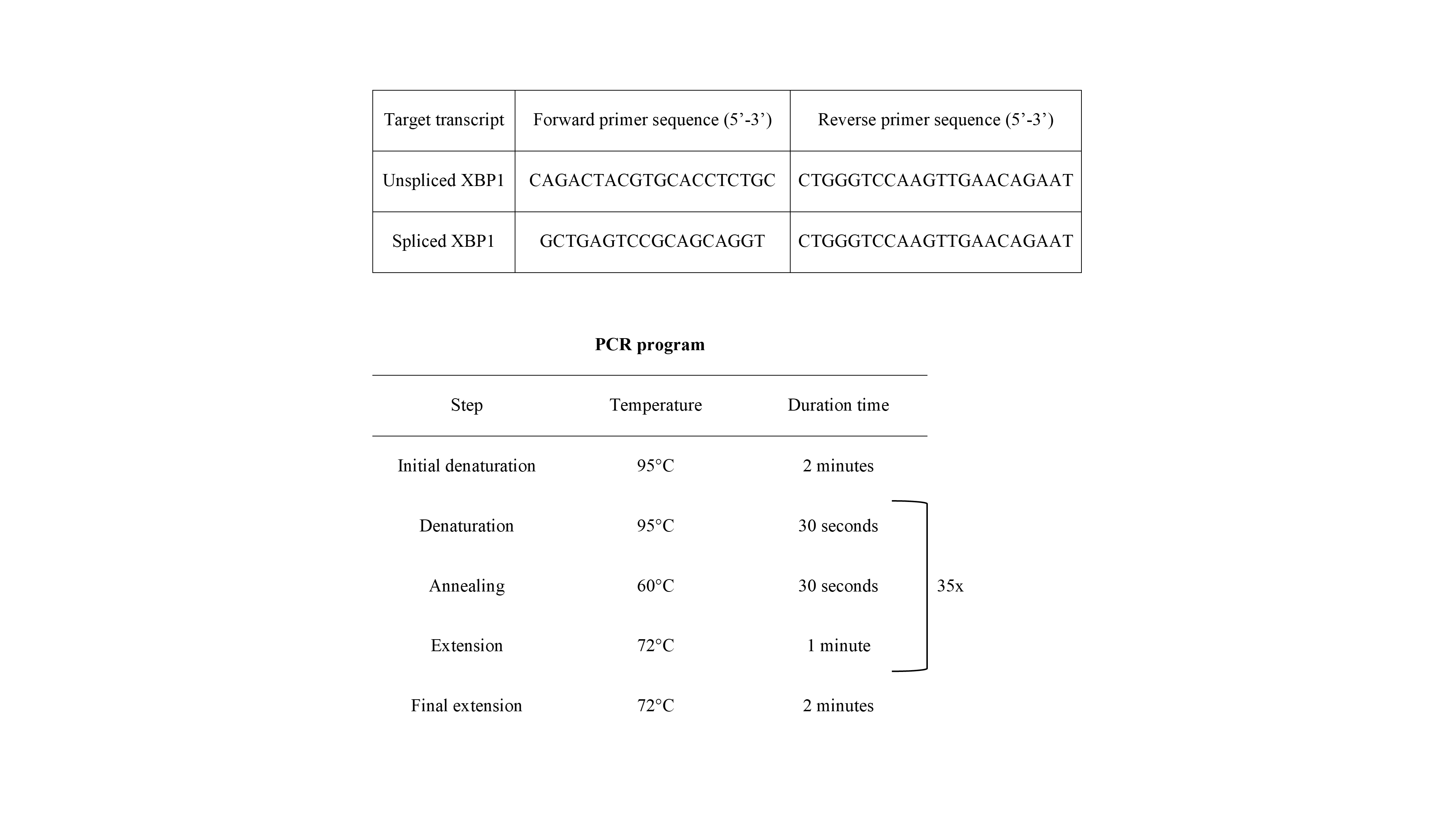

Supplement: S1 Table — (TIF) [file pone.0260123.s001.tif]

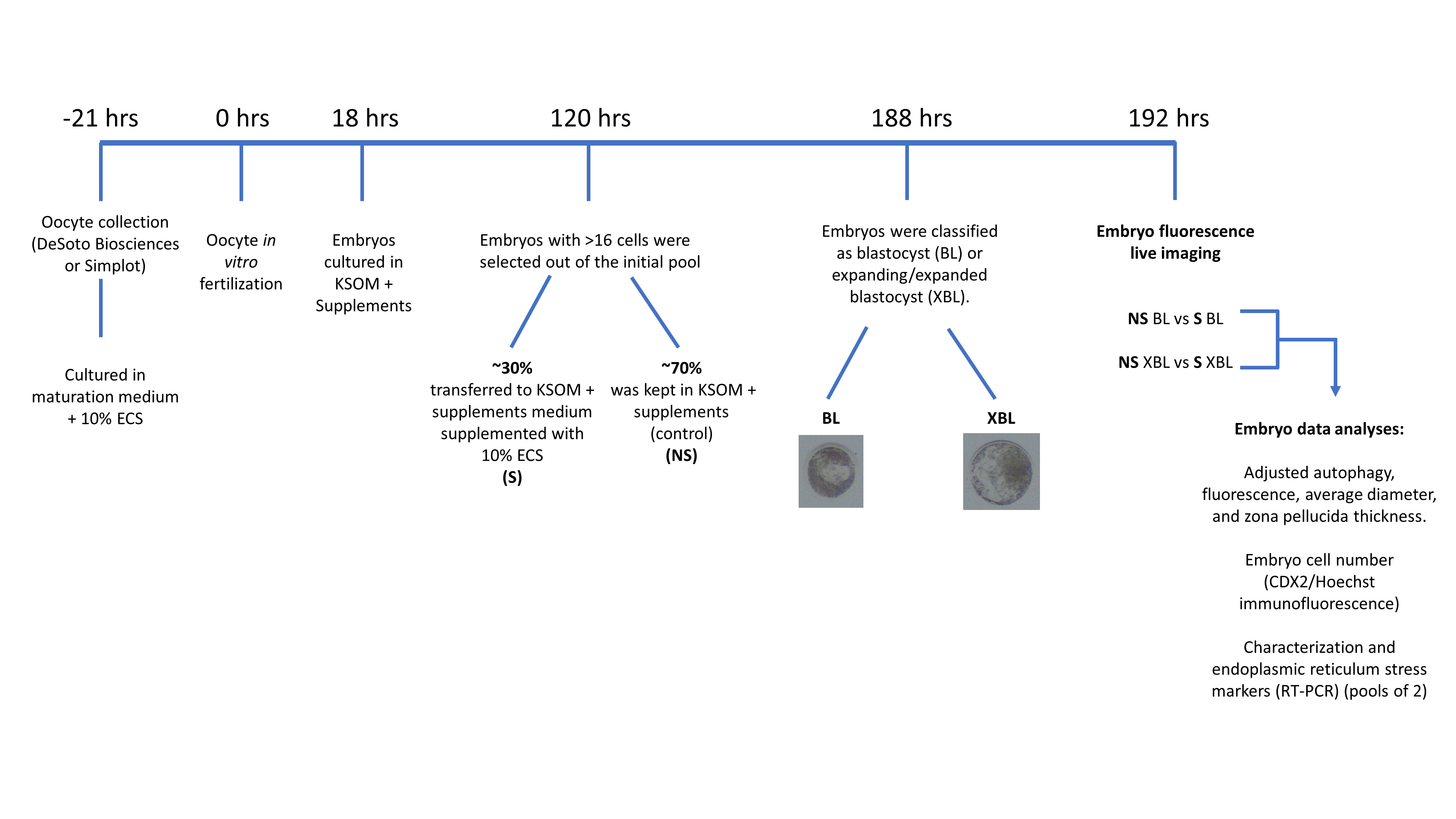

Supplement: S1 Fig — (TIF) [file pone.0260123.s002.tif]

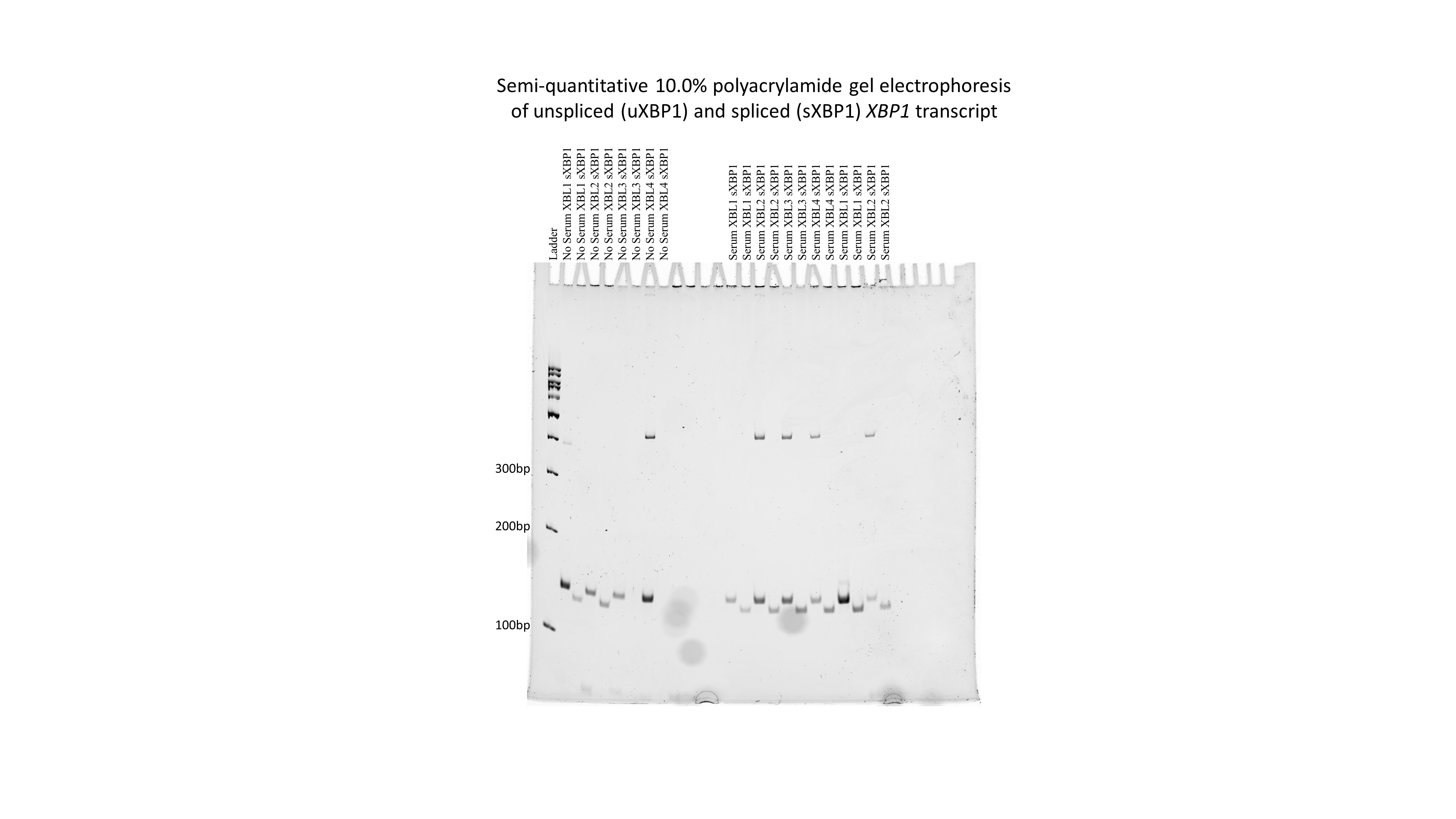

Supplement: S2 Fig — A. Unspliced, spliced, and total XBP1 ratios of single bovine embryos. B. Ratio of spliced XBP1 of tunicamycin treated, vehicle, and control embryos. TM treatment indeed shows splicing of XBP1, suggesting an UPR response to induced ER stress. TM = 5μg Tunicamycin. Vehicle = 0.05% ethanol alcohol. (TIF) [file pone.0260123.s003.tif]
