# Supplementary material for: Serum supplementation during bovine embryo culture affects their development and proliferation through macroautophagy and endoplasmic reticulum stress regulation
Source: PLoS One. 2021 Dec 9;16(12):e0260123. doi: 10.1371/journal.pone.0260123 (PMC8659681; doi:10.1371/journal.pone.0260123)
Supplement: S1 Raw images — (PDF) [file pone.0260123.s004.pdf]

Oligo primer and PCR optimization information

9/10/20

Bovine	
Gene Name	XBP1
Assembly	ARS-UCD 1.2
Accession Number	NM_001034727.3
PMID	31329612
Molecule	mRNA
Made by	Edgar Soto-Moreno
Optimized by	Edgar Soto-Moreno
Order Date	1/Jun/2020

Notes:
Primer amplifies unspliced form of XBP1 transcript (mRNA).

Edgar Soto-Moreno		Page (s)
Notebook	2	34-35
Binder	1	290

Primer Name		Sequence (5'-3')
BEuXBP1NM_001034727.3EJSM_F1	Forward	CAGACTACGTGCACCTCTGC
BEuXBP1NM_001034727.3EJSM_R1	Reverse	CTGGGTCCAAGTTGAACAGAAT

Components	Final [ ]	Master Mix/sample
Nuclease Free Water	-	11.3ul
Go Taq Flexi Buffer [5x] Promega M890A	1X	4ul
MgCl2 [25mM] Promega A351H	2.5mM	2ul
dNTPs [10mM] Sigma D7295	0.2mM	0.4ul
Forward Primer [10uM]	0.3uM	0.6ul
Reverse Primer [10uM]	0.3uM	0.6ul
Go Taq Flexi Polymerase Promega M8295	1 unit	0.1ul
Template [~25ng/ul]	-	1ul

PCR Program

Initial Denaturation 95°C for 2min
Denaturation 95°C for 30sec
Annealing 60.2°C for 30sec
Initial Extension 72°C for 1min
35 Cycles
Final Extension 72°C for 2min
(Ramping: Max)

Samples:
cDNA
Day 105 fetal muscle (K189)

Negative Control:
Nuclease Free Water
-RT

Ladder:
Catalog # NEB N3231S
6 uL (1Ladder: 1Dye: 4H2O)
100bp -1,000bp
Stored at 4°C fridge

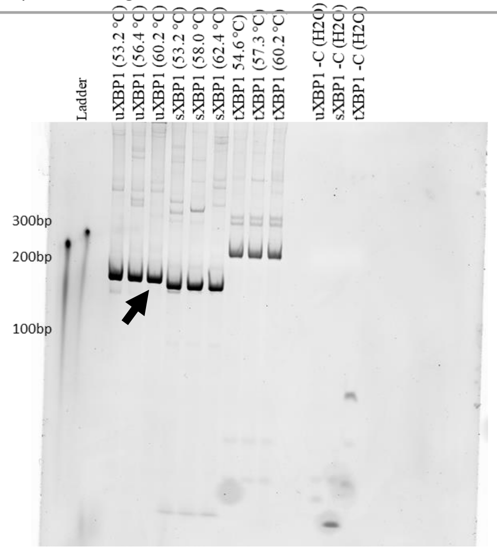

Amplicon Sequence:

CAGACTACGTGCACCTCTGCAGCAGGTGCAGGCCCAGTTGT
CACCCCTCCAGAACATCTCCCCATGGACTCTGATGGCATTGA
CTCTTCAGACTCTGAGTCTGACATCCTGTT**GGGCATTCTGTT**
CAACTTGGACCCAG

uXBP1 Amplicon size: 139bp

Gel notes: 7% PAGE, 2 hours @ 200 Volts
Ladder did not appear/migrate properly. Used migration of bromophenol blue (~52bp) and xylene cyanol (~190bp) to estimate amplicon size/migration.

9/10/20

Bovine	
Gene Name	XBP1
Assembly	ARS-UCD 1.2
Accession Number	NM_001271737.1
PMID	31329612
Molecule	mRNA
Made by	Edgar Soto-Moreno
Optimized by	Edgar Soto-Moreno
Order Date	1/Jun/2020

Notes:
Primer amplifies spliced form of XBP1 transcript (mRNA).

Edgar Soto-Moreno		Page (s)
Notebook	2	34-35
Binder	1	290

Primer Name		Sequence (5'-3')
BEsXBP1NM_001271737.1EJSM_F1	Forward	GCTGAGTCCGCAGCAGGT
BEsXBP1NM_001271737.1EJSM_R1	Reverse	CTGGGTCCAAGTTGAACAGAAT

Components	Final [ ]	Master Mix/sample
Nuclease Free Water	-	11.3ul
Go Taq Flexi Buffer [5x]<br>Promega M890A	1X	4ul
MgCl2 [25mM]<br>Promega A351H	2.5mM	2ul
dNTPs [10mM]<br>Sigma D7295	0.2mM	0.4ul
Forward Primer [10uM]	0.3uM	0.6ul
Reverse Primer [10uM]	0.3uM	0.6ul
Go Taq Flexi Polymerase<br>Promega M8295	1 unit	0.1ul
Template [~25ng/ul]	-	1ul

PCR Program

Initial Denaturation 95°C for 2min
Denaturation 95°C for 30sec
Annealing 60.2°C for 30sec
Initial Extension 72°C for 1min
35 Cycles
Final Extension 72°C for 2min
(Ramping: Max)

Samples:
cDNA
Day 105 fetal muscle (K189)
Negative Control:
Nuclease Free Water
-RT

Ladder:
Catalog # NEB N3231S
6 uL (1Ladder: 1Dye: 4H2O)
100bp -1,000bp
Stored at 4°C fridge

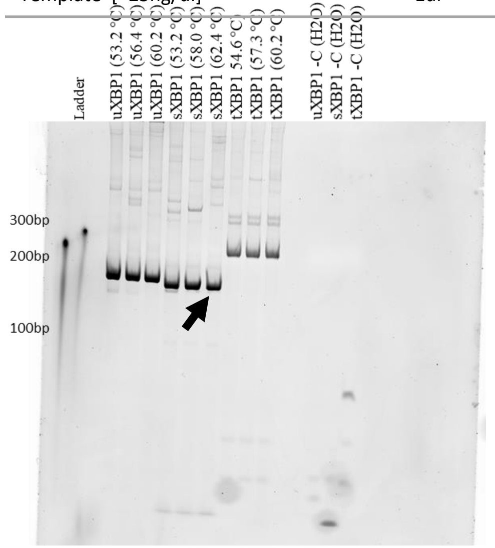

Amplicon Sequence:

GCTGAGTCCGCAGCAGGTGCAGGCCCAGTTGTCACCCCTCC AGAACATCTCCCCATGGACTCTGATGGCATTGACTCTTCAGA CTCTGAGTCTGACATCCTGTTGGGC**ATTCTGTTCAACTTGGA CCCAG**

sXBP1 Amplicon size: 130bp

Gel notes: 7% PAGE, 2 hours @ 200 Volts
Ladder did not appear/migrate properly. Used migration of bromophenol blue (~52bp) and xylene cyanol (~190bp) to estimate amplicon size/migration.

Original gel pictures and additional information

Day 5 embryo pools RT-PCR for unspliced (u) and spliced (s) XBP1 (ER stress marker) (10.0% acrylamide)- 1/18/2021

NS = no serum
S = 10% serum
TM = Tunicamycin

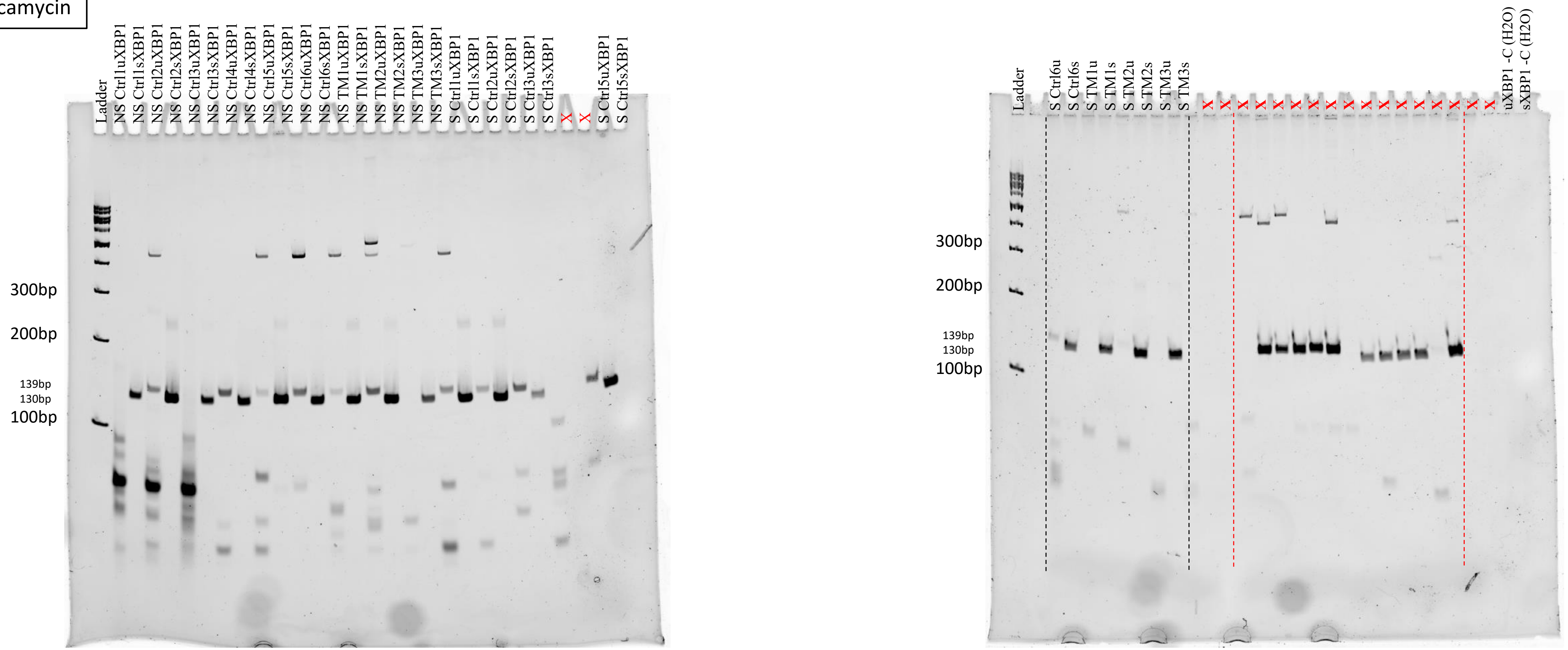

8.5sec manual exposure

XBP1 mRNA and its ER stress-dependent spliced form analysis was done by semi-quantitative RT-PCR (S2 Fig). Previously published intron-spanning oligo primers for spliced and unspliced XBP1 isoforms were used in order to minimize bias created by potential DNA contamination (41) (S1 table). In the aforementioned report, the forward primer for each *XBP1* variant was designed to bind specifically to either the unspliced or spliced exon 4 sequence, while the same reverse primer was used for both products. RT-PCR products were resolved in a 10% polyacrylamide gel (S2 Fig). Images were taken during 8.5 seconds of UV light exposure in a QuantStudio ChemiDoc system (BioRad). Total *XBP1* levels (unspliced + spliced) were used to normalize the expression and percentage of spliced *XBP1* mRNA and band density (including background intensity) was quantified using FIJI software. These gels were used to generate Figure 5C.

NS = no serum
S = 10% serum
BL = blastocyst
XBL = expanding/expanded blastocyst

Single embryo RT-PCR for unspliced and spliced XBP1 (ER stress marker) (10.0% acrylamide)-
4/19/2021

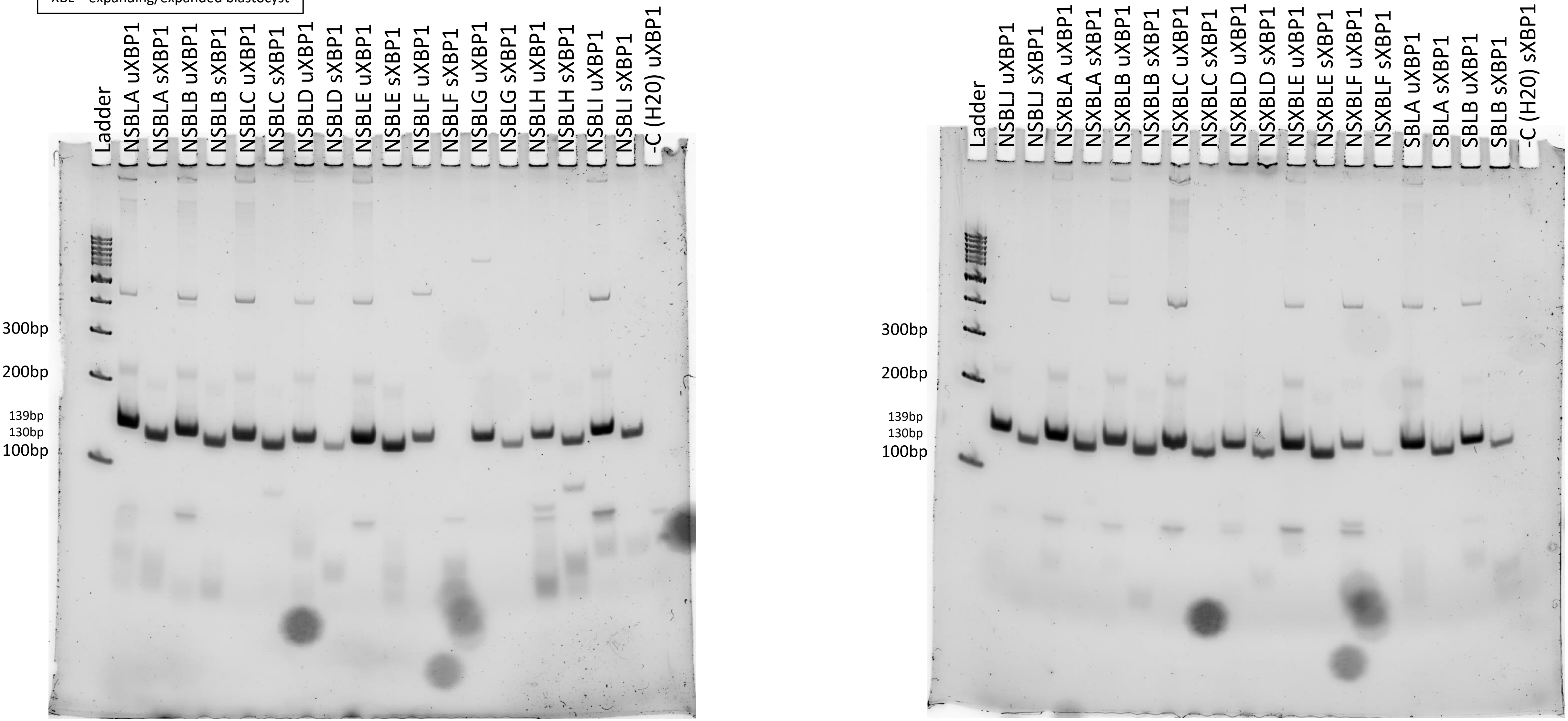

XBP1 mRNA and its ER stress-dependent spliced form analysis was done by semi-quantitative RT-PCR (S2 Fig). Previously published intron-spanning oligo primers for spliced and unspliced XBP1 isoforms were used in order to minimize bias created by potential DNA contamination (41) (S1 table). In the aforementioned report, the forward primer for each *XBP1* variant was designed to bind specifically to either the unspliced or spliced exon 4 sequence, while the same reverse primer was used for both products. RT-PCR products were resolved in a 10% polyacrylamide gel (S2 Fig). Images were taken during 8.5 seconds of UV light exposure in a QuantStudio ChemiDoc system (BioRad). Total *XBP1* levels (unspliced + spliced) were used to normalize the expression and percentage of spliced *XBP1* mRNA and band density (including background intensity) was quantified using FIJI software. These gels were used to generate Figure 5D.

NS = no serum
S = 10% serum
BL = blastocyst
XBL = expanding/expanded blastocyst

Single embryo RT-PCR for unspliced and spliced XBP1 (ER stress marker) (10.0% acrylamide)-
4/19/2021

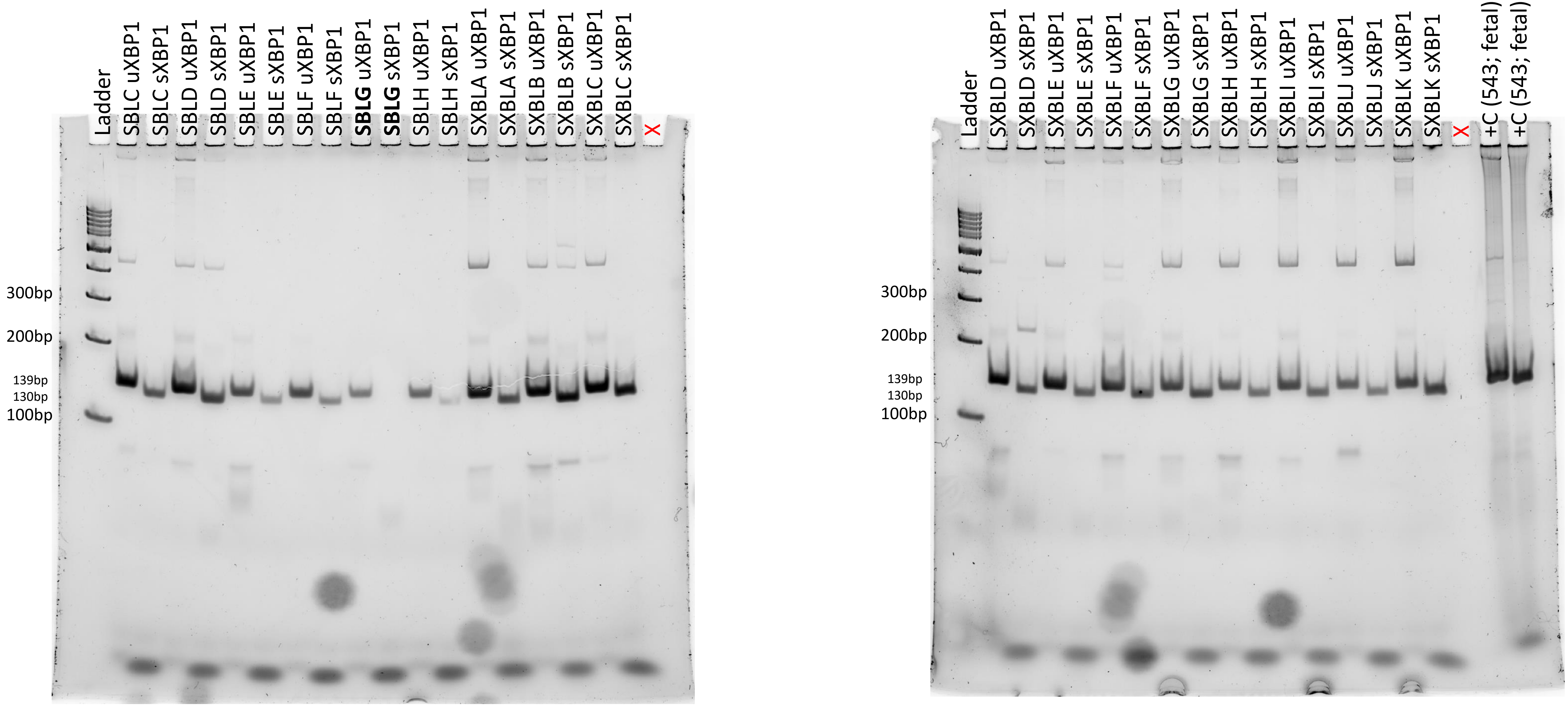

XBP1 mRNA and its ER stress-dependent spliced form analysis was done by semi-quantitative RT-PCR (S2 Fig). Previously published intron-spanning oligo primers for spliced and unspliced XBP1 isoforms were used in order to minimize bias created by potential DNA contamination (41) (S1 table). In the aforementioned report, the forward primer for each *XBP1* variant was designed to bind specifically to either the unspliced or spliced exon 4 sequence, while the same reverse primer was used for both products. RT-PCR products were resolved in a 10% polyacrylamide gel (S2 Fig). Images were taken during 8.5 seconds of UV light exposure in a QuantStudio ChemiDoc system (BioRad). Total *XBP1* levels (unspliced + spliced) were used to normalize the expression and percentage of spliced *XBP1* mRNA and band density (including background intensity) was quantified using FIJI software. These gels were used to generate Figure 5D.
